# CD1d expression in glioblastoma is a promising target for NKT cell-based cancer immunotherapy

**DOI:** 10.1007/s00262-020-02742-1

**Published:** 2020-10-31

**Authors:** Ayaka Hara, Ryo Koyama-Nasu, Mariko Takami, Takahide Toyoda, Takahiro Aoki, Fumie Ihara, Masayoshi Kobayashi, Seiichiro Hirono, Tomoo Matsutani, Toshinori Nakayama, Yasuo Iwadate, Shinichiro Motohashi

**Affiliations:** 1 Department of Medical Immunology, Graduate School of Medicine, Chiba University, 1-8-1 Inohana, Chuo-ku, Chiba, 260-8670 Japan; 2 Department of Neurological Surgery, Graduate School of Medicine, Chiba University, Chiba, Japan; 3 Department of Immunology, Graduate School of Medicine, Chiba University, Chiba, Japan

**Keywords:** Glioblastoma, NKT cells, CD1d, Stem-like cell, Retinoic acid, Cancer immunotherapy

## Abstract

Glioblastoma is the most common and aggressive type of brain tumor with high recurrence and fatality rates. Although various therapeutic strategies have been explored, there is currently no effective treatment for glioblastoma. Recently, the number of immunotherapeutic strategies has been tested for malignant brain tumors. Invariant natural killer T (iNKT) cells play an important role in anti-tumor immunity. To address if iNKT cells can target glioblastoma to exert anti-tumor activity, we assessed the expression of CD1d, an antigen-presenting molecule for iNKT cells, on glioblastoma cells. Glioblastoma cells from 10 of 15 patients expressed CD1d, and CD1d-positive glioblastoma cells pulsed with glycolipid ligand induced iNKT cell-mediated cytotoxicity in vitro. Although CD1d expression was low on glioblastoma stem-like cells, retinoic acid, which is the most common differentiating agent, upregulated CD1d expression in these cells and induced iNKT cell-mediated cytotoxicity. Moreover, intracranial administration of human iNKT cells induced tumor regression of CD1d-positive glioblastoma in orthotopic xenografts in NOD/Shi-scid IL-2RγKO (NOG) mice. Thus, CD1d expression represents a novel target for NKT cell-based immunotherapy for glioblastoma patients.

**Electronic supplementary material:**

The online version of this article (10.1007/s00262-020-02742-1) contains supplementary material, which is available to authorized users.

## Introduction

Glioblastoma is the most common form of a malignant primary brain tumor in adults. Despite recent advances in multimodal therapies that combine maximal surgical resection with postoperative adjuvant chemoradiotherapy, median survival is only around 15–16 months, and 5-years survival is less than 10% [1, 2]. Therefore, there is an urgent need to develop better treatments for glioblastoma.

Small subpopulations of neoplastic cells with stem-like properties have been identified in leukemia and solid tumors including glioblastoma [3, 4]. Glioblastoma stem-like cells grown in serum-free media as neurospheres have the capabilities of self-renewal, differentiation, and tumorigenicity [5, 6].

Cancer immunotherapy is a potential therapeutic option for glioblastoma [7]. Recent progress in the development of immune checkpoint inhibitors, such as anti-programmed cell death-1 (PD-1) antibodies and chimeric antigen receptor (CAR) T cells, holds promise for the treatment of various cancer types [8]. However, in the case of glioblastoma, a large randomized phase III study evaluating nivolumab, an inhibitory antibody against PD-1, recently failed to demonstrate a survival benefit compared with bevacizumab in glioblastoma patients with first or second recurrence [9, 10]. CAR T cell immunotherapy, an adoptive T cell therapy, has been studied intensively for glioblastoma [11, 12]. Christine et al. reported that the regression of all intracranial and spinal tumors was observed, along with corresponding increases in the levels of cytokines and immune cells in the cerebrospinal fluid, after intracranial infusions of IL13Rα2-targeted CAR T cells [13].

Invariant natural killer T (iNKT) cells belong to a unique lineage of innate lymphocytes characterized by restricted T cell receptor (TCR) usage (Vα24/Vβ11 in humans). iNKT cells recognize glycolipid ligands presented on CD1d molecules and respond to a synthetic glycolipid, α-galactosylceramide (α-GalCer) [14]. Importantly, iNKT cells are known to play a role in anti-tumor immunity [15]. Indeed, α-GalCer-pulsed antigen-presenting cells in patients with advanced or recurrent non-small cell lung cancer elicited iNKT cell-dependent immune responses, which were correlated with prolonged overall survival time [16].

CD1d is preferentially expressed in normal and malignant hematopoietic cells, especially those of myelomonocytic and B cell lineages [17–19]. Although the majority of solid tumors are CD1d negative, CD1d expression by several glioblastoma cell lines has been reported [20]. However, glioblastoma has not been recognized as a target for immunotherapy with iNKT cells.

In this study, we confirmed the expression of CD1d in surgically resected fresh specimens from 10 of 15 glioblastoma patients. Furthermore, CD1d-positive glioblastoma cells were sensitive to direct iNKT cell-mediated cytotoxicity with glycolipid antigens. Moreover, the intracranial administration of human iNKT cells led to the regression of established orthotropic human CD1d-positive glioblastoma xenografts in NOD/Shi-scid IL-2RγKO (NOG) mice.

## Materials and methods

### Patients

Peripheral blood mononuclear cells (PBMCs) and surgically resected tumor specimens were obtained from glioblastoma patients (mean age of 61 years; range 48–73 years). Patients met the histological criteria for a diagnosis of glioblastoma and were treated at the Department of Neurological Surgery, Chiba University Hospital, Japan. Informed consent was obtained from all the patients. The protocol was approved by the Institutional Ethics Committee.

### Preparation of tumor-infiltrating lymphocytes (TILs) and tumor cells

Surgically resected fresh tumor tissues were mechanically minced and digested in RPMI 1640 medium with an enzyme mixture from a Tumor Dissociation Kit, human (Miltenyi Biotec, Bergisch Gladbach, Germany) using a gentleMACS Dissociator. The resultant cell suspension was washed in Hanks’ Balanced Salt Solution (HBSS) and subjected to two-layered separation (75% and 100%) by discontinuous density gradient centrifugation in Lymphocyte Separation Media (MP Biomedicals, Santa Ana, CA). The cells from the 100% and 75% interfaces were used as TILs and tumor cells.

### Flow cytometry

iNKT cells were identified by staining with fluorochrome-conjugated monoclonal antibodies against TCR Vα24 (C15; Beckman Coulter, Brea, CA), TCR Vβ11 (C21; Beckman Coulter), and CD3ε (UCTH1; BD Biosciences, Franklin Lakes, NJ). CD1d expression on tumor cells was evaluated by staining with a monoclonal antibody against CD1d (51.1; Thermo Fisher Scientific, Waltham, MA). Data were collected on the FACS Canto™ II (BD Biosciences) and analyzed using the FLOWJO (FlowJo LLC) software program. Data normalization of CD1d expression on tumor cells was performed by transforming the median fluorescence intensity (MFI) values of the test samples to a common scale using the following equation: Final relative MFI = MFI of the test sample/MFI of the isotype control.

### Cell lines and culture conditions

Human brain tumor cell lines (U87, U138, U251, and T98G) were purchased from the American Type Culture Collection (Manassas, VA, USA). These cell lines were cultured in DMEM (Thermo Fisher Scientific) supplemented with 10% fetal bovine serum and antibiotics under a humidified atmosphere containing 5% CO_2_ at 37 °C. Patient glioblastoma cells were first cultured in DMEM/F-12 medium (Thermo Fisher Scientific) containing B27 supplement minus vitamin A (Thermo Fisher Scientific), epidermal growth factor, and basic fibroblast growth factor (20 ng/ml each; Wako Pure Chemical Industries, Osaka, Japan) to generate glioblastoma stem-like cells as neurospheres. These glioblastoma stem-like cells were then cultured in DMEM supplemented with 10% fetal bovine serum (FBS) to generate patient-derived glioblastoma cell lines or with all-trans retinoic acid (RA) for differentiation. To generate CD1d-transfected U87 cells, pCMV6-XLA4/hCD1d (OriGene Technologies, Rockville, MD, USA) was transfected into U87 cells using a cationic lipid-based transfection reagents, Lipofectamine 3000 (Thermo Fisher Scientific), according to the respective manufacturer’s instructions. Mock group cells were mock-transfected with Lipofectamine 3000 reagent only.

### iNKT cell expansion and sorting

PBMCs from healthy donors were isolated by density gradient separation using Ficoll-Paque™ PLUS (GE Healthcare, Chicago, IL, USA). PBMCs were cultured in RPMI 1640 complete medium with α-GalCer (KRN7000; REGiMMUNE, Tokyo, Japan) and IL-2 (Imunace; Shionogi, Osaka, Japan) to expand iNKT cells for 8 − 12 days. iNKT cells labeled with FITC-conjugated anti-Vα24 antibody were positively sorted using Anti-FITC MicroBeads (Miltenyi Biotec). Sorted iNKT cells were maintained in RPMI 1640 complete medium with IL-2 for 2–3 days and were used for the cytotoxicity assays and cytokine quantification assays.

### RNA isolation and real-time RT-PCR

Total RNA was extracted from cell lines and patient tumor cells using an RNeasy Mini Kit (Qiagen, Venlo, Netherlands) and reverse-transcribed using SuperScript IV VILO Master Mix (Thermo Fisher Scientific). Real-time PCR was performed using a Universal ProbeLibrary System (Roche, Basel, Switzerland) and an Applied Biosystems 7500Fast sequence detector (Thermo Fisher Scientific). Gene expression was normalized against *GAPDH* expression.

### Cytotoxicity assay

Tumor cells were cultured overnight in medium containing α-GalCer or vehicle control. iNKT cells were co-cultured with α-GalCer-pulsed tumor cells at effector-to-target (E:T) ratios. Cell apoptosis was evaluated by staining with annexin V and 7-AAD (BD Biosciences).

Cytotoxicity was also assessed by measuring lactate dehydrogenase (LDH) release. The LDH detection assay was performed using an LDH Cytotoxicity Detection Kit (Roche, Mannheim, Germany) according to the manufacturer’s recommendations.

### Cytokine quantification assay

Cytokine production by iNKT cells was assessed through co-culture with target tumor cells at a 5:1 ratio. The target tumor cells were cultured overnight in medium containing α-GalCer or vehicle control. After 24 h, cytokine levels were measured. Cytokines produced by iNKT cells were evaluated by CBA Plex beads on a FACS Array bioanalyzer (BD Biosciences).

### Orthotopic xenograft model of glioblastoma in NOG mice

All animal experiments were approved by the Chiba University Review Board for Animal Care. All mice were maintained under specific pathogen-free conditions. NOG mice were obtained from In-Vivo Science Inc. (Kawasaki, Japan). Male 6–8-week-old mice were anesthetized and immobilized in a stereotactic apparatus fitted for animal experimentation. The injection coordinates were 2 mm to the right of the midline, 1 mm anterior to the coronal suture, and 3.5-mm deep. Glioblastoma cells (1 × 10^5^), iNKT cells (1 × 10^6^), α-GalCer (200 ng), or vehicle control were injected 3.5-mm deep using a Hamilton syringe (Hamilton, Reno, NV, USA). Mice were monitored for 3 months.

### In vivo micro-CT imaging

All mice were scanned up to 6 weeks after engraftment. Directly before image acquisition, an iodine-based contrast agent (Iomeprol; Imeron 300, Bracco Imaging Group, Milano, Italy) was injected via the lateral tail vein under general anesthesia. Tumor diameter of micro-CT images was measured according to the response assessment in neuro-oncology (RANO) criteria, which are used to evaluate response to first-line treatment for glioblastoma [21]. The product of maximal diameter and second perpendicular measurement was determined.

### Bioluminescence imaging

Bioluminescence images were collected on an IVIS Lumina II Imaging System (PerkinElmer, Waltham, MA) using Living Image, a software program provided by the same manufacturer. All mice were anesthetized and received a single intraperitoneal injection of 150 mg/kg D-luciferin (Promega KK, Madison, WI, USA) 10 min before scanning. All mice were imaged weekly.

### Statistical analysis

Statistical analysis was performed using the JMP13 (SAS Institute Inc., Cary, NC, USA). The survival curves were determined using the Kaplan–Meier method. The log-rank test was used for comparison. A *P *value of < 0.05 was considered statistically significant.

## Results

### CD1d expression in patient glioblastoma cells

**Fig. 1:**
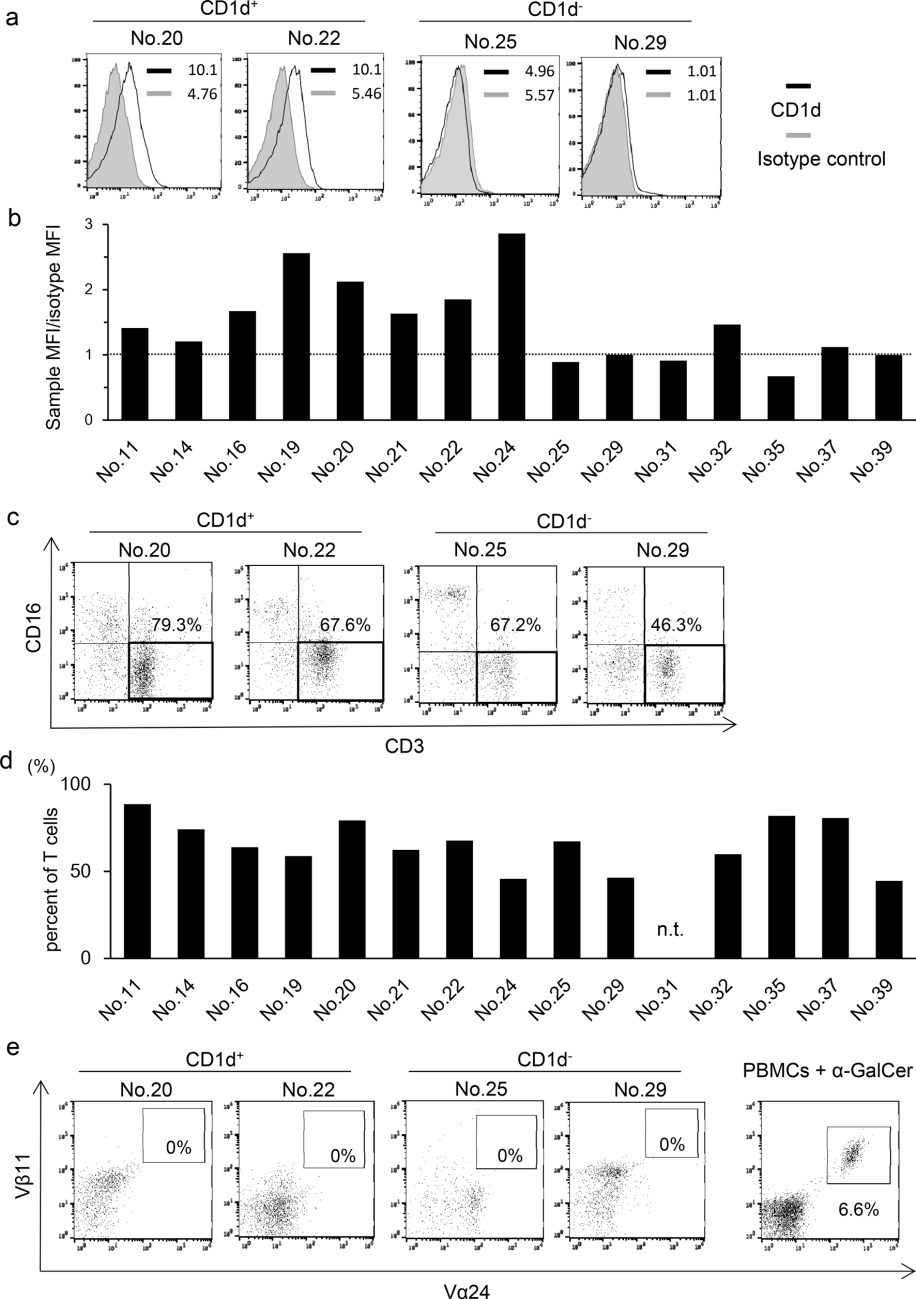
CD1d expression in patient glioblastoma cells and CD3^+^ T cell and Vα24^+^ iNKT cell frequency in TILs. **a** Expression levels of CD1d in patient glioblastoma cells were evaluated by flow cytometry. Hematopoietic cells were excluded by CD45 staining. Representative histograms of two CD1d-positive and two CD1d-negative patients are shown. MFI is indicated for both isotype and anti-CD1d staining. Solid lines: staining profiles of CD1d. Shaded areas: background staining with an isotype control. **b** Bar graph represents the expression levels of CD1d in patient glioblastoma cells. The *x*-axis represents individual glioblastoma patients and the *y*-axis represents relative MFI of CD1d. The dotted line indicates relative MFI 1.0. **c** The proportion of CD3^+^ T cells in patient TILs was evaluated by flow cytometry. Representative histograms of two CD1d-positive and two CD1d-negative patients are shown. Values represent the percentages of CD3^+^ T cells in patient TILs. **d** The bar graph represents infiltration of CD3^+^ T cells in TILs which was CD45 positive. n.t.: not tested. **e** The frequency of Vα24^+^ iNKT cells in patient TILs was assessed as described in **c**. Values represent the percentages of Vα24^+^ iNKT cells in TILs. Right-hand panel indicates PBMCs treated with α-GalCer as a positive control

As the activation of iNKT cells is CD1d-restricted, we first examined CD1d expression in patient glioblastoma cells. We performed flow cytometric analysis of surgically resected patient glioblastoma tissue and found that tumor cells from some patients expressed CD1d on the cell surface (Fig. 1a, b). Ten out of fifteen patients (66.7%) were CD1d positive, with a value of more than 1.0 for the relative MFI (Fig. 1b).

### Frequency of T cells and iNKT cells in TILs

T lymphocytes play a pivotal role in anti-tumor immunity. We next performed flow cytometric analysis of TILs from glioblastoma patients. The profiles of both CD1d-positive and -negative patients were investigated. Infiltration of CD3^+^ T cells into brain tumor tissue was observed in 14 patients (Fig. 1c, d), however, infiltration of Vα24^+^ iNKT cells was not detected in any patient (Fig. 1e). These results suggested that iNKT cells were unable to infiltrate glioblastoma irrespective of their CD1d expression.

### CD1d expression in human glioblastoma cell lines

**Fig. 2:**
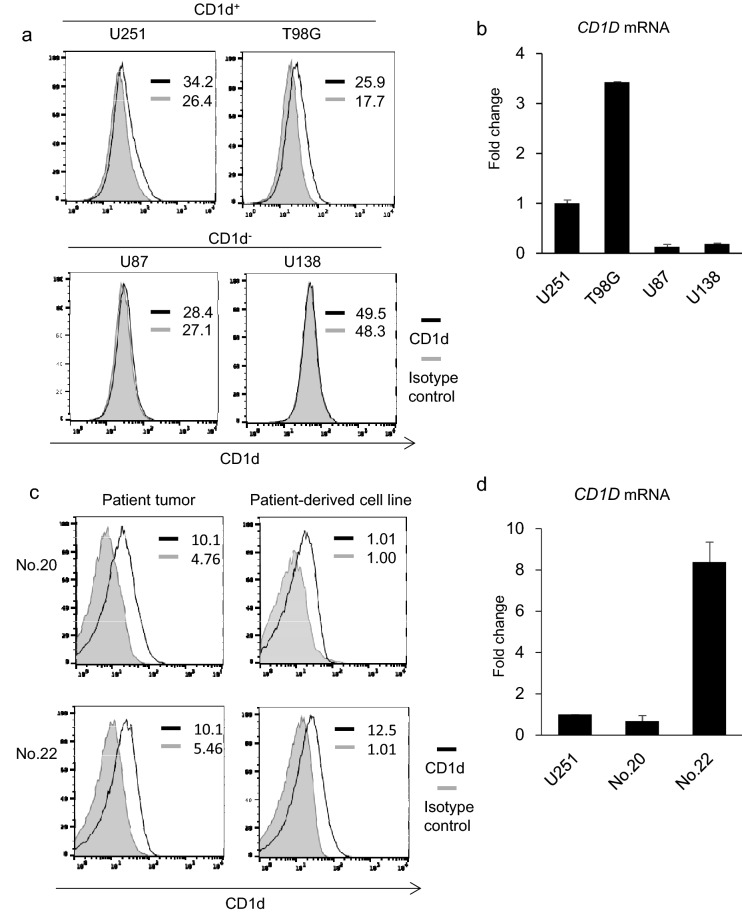
CD1d expression in glioblastoma cell lines and patient-derived glioblastoma cell lines. **a** The expression levels of CD1d in glioblastoma cell lines were assessed by flow cytometry. MFI is indicated for both isotype and anti-CD1d staining. Solid lines: staining profiles of CD1d. Shaded areas: background staining with an isotype control. **b** The expression levels of *CD1D* mRNA in glioblastoma cell lines were analyzed by quantitative RT-PCR and shown as fold change over mRNA levels in U251 cells. Error bars represent the standard deviation (*n* = 3). **c** The expression levels of CD1d in glioblastoma cell lines generated from the indicated patients were assessed as described in **a**. **d** The expression levels of *CD1D* mRNA in glioblastoma cell lines generated from the indicated patients were analyzed as described in **b**

Many cell lines derived from malignant glioma have been established and widely used as model systems. Therefore, we examined CD1d expression in four human glioblastoma cell lines by flow cytometry. Two lines, U251 and T98G, were found to express CD1d on the cell surface and the relative MFI was more than 1.0. Conversely, U87 and U138 did not express detectable levels of CD1d and the relative MFI was less than or equal to 1.0 (Fig. 2a). We next examined whether the levels of the cell surface expression of CD1d were associated with *CD1D* mRNA levels. RT-PCR analysis of human glioblastoma cell lines confirmed CD1d expression in both U251 and T98G cell lines but not in U87 or U138 cells, suggesting that CD1d expression in glioblastoma cells was mainly regulated at the transcriptional level (Fig. 2b).

### CD1d expression in patient-derived glioblastoma cell lines

To further investigate if cell lines can reflect the gene expression properties of the originating patient glioblastoma tissue, we established cell lines from CD1d-positive patient glioblastoma samples. Similar levels of CD1d expression were ascertained in the original patient glioblastoma tissue and patient-derived cell lines (Fig. 2c). Consistent with this notion, the mRNA levels of the *CD1D* gene in CD1d-positive patient-derived cell lines were comparable with the levels in U251 cells (Fig. 2d). Thus, glioblastoma cell lines from CD1d-positive patient glioblastoma samples maintain the status of CD1d expression from original patient tumor tissue, and can be used for the evaluation of iNKT cell-mediated cytotoxicity toward glioblastoma cells.

### iNKT cell-mediated cytotoxicity toward glioblastoma cells

**Fig. 3:**
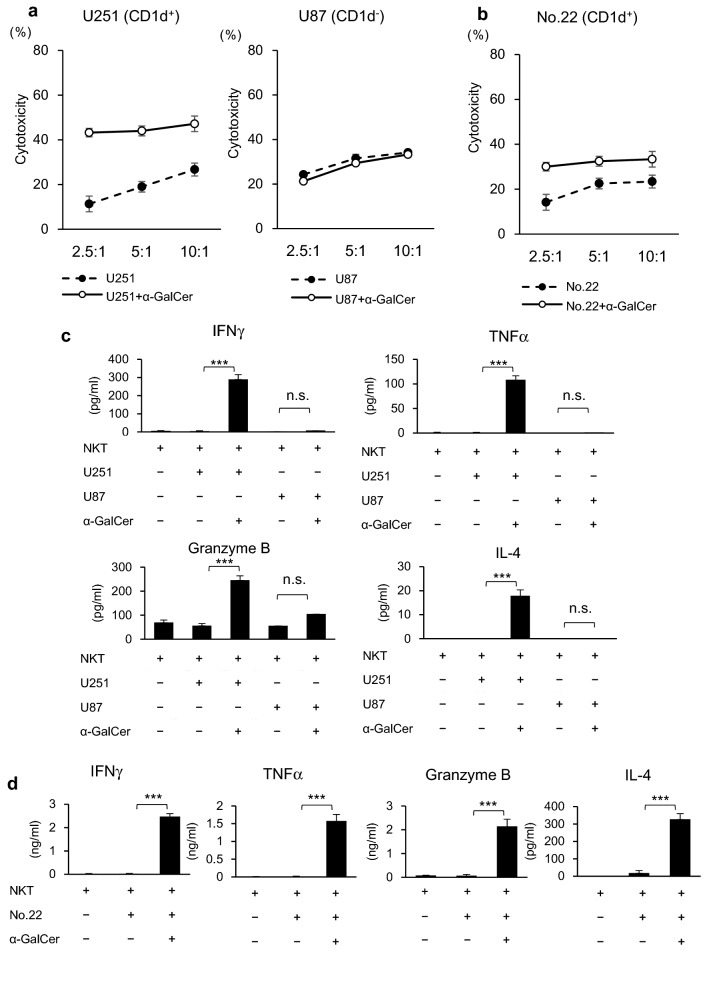
iNKT cell-mediated cytotoxicity and cytokine response towards CD1d-positive glioblastoma cells. **a** Glioblastoma cell lines were pulsed overnight with α-GalCer (200 ng/ml) and co-cultured with resting iNKT cells for 4 h at the indicated E:T ratios. Cell apoptosis was evaluated by staining with annexin V and 7-AAD. Error bars represent the standard error of the mean (SEM) (*n* = 3). **b** Patient-derived cell line No.22 was analyzed as described in **a**. **c** Glioblastoma cell lines were pulsed overnight with α-GalCer (200 ng/ml) and co-cultured with resting iNKT cells for 24 h. Production of IFNγ, TNFα, granzyme B, and IL-4 were measured by CBA multiplex assay. Error bars represent the standard deviation (*n* = 3). ***P < 0.001. *n.s.* not significant. **d** Patient-derived cell line No.22 was analyzed as described in **c**

Since direct cytotoxicity is one of the most important functions of activated iNKT cells, we performed an in vitro cytotoxicity assay using CD1d-positive and -negative human glioblastoma cell lines. To assess whether α-GalCer could improve iNKT cell-mediated cytotoxicity, tumor cells were pulsed with α-GalCer overnight and cytotoxicity assays were performed with purified iNKT cells at E:T ratios of 2.5:1, 5:1, and 10:1. iNKT cells were only found to be highly cytotoxic against α-GalCer-pulsed CD1d-positive glioblastoma cell line U251 (Fig. 3a). In contrast, iNKT cells showed a minimal effect on CD1d-negative U87 cells irrespective of α-GalCer treatment (Fig. 3a). Furthermore, CD1d-positive patient-derived cell line No. 22 was also sensitive to α-GalCer-dependent iNKT cell-mediated cytotoxicity (Fig. 3b).

### Cytokine production of iNKT cells with glioblastoma cells

We then evaluated the cytokine production of iNKT cells when stimulated with CD1d-positive and -negative human glioblastoma cells. Purified iNKT cells were co-cultured with human glioblastoma cell lines that had been pulsed overnight with α-GalCer or vehicle control, and the cytokine concentrations in the culture supernatant were measured. Interferon-γ (IFNγ), tumor necrosis factor-α (TNFα), granzyme B, and IL-4 levels were significantly increased in iNKT cells stimulated with the α-GalCer-pulsed glioblastoma cell lines in a CD1d-dependent manner (Fig. 3c). Consistent with this result, CD1d-positive patient-derived cell line No. 22 could promote a cytokine response in iNKT cells (Fig. 3d). Altogether, these results indicated that CD1d expression in glioblastoma triggered iNKT cell-mediated anti-tumor responses.

### RA induces CD1d expression in stem-like cells derived from patient glioblastoma

**Fig. 4:**
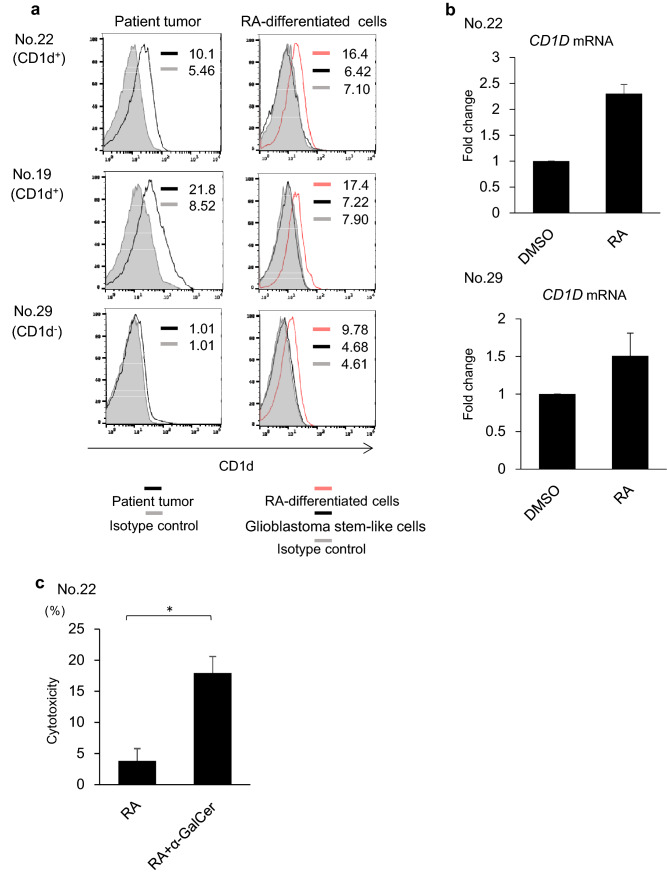
RA induces glioblastoma stem-like cell CD1d expression. **a** The expression levels of CD1d in glioblastoma stem-like cells and RA-differentiated cells were assessed by flow cytometry. MFI is indicated for both isotype and anti-CD1d staining. Solid lines: staining profiles of CD1d. Shaded areas: background staining with an isotype control. Red lines: CD1d staining profiles of RA-differentiated cells. **b** The expression levels of *CD1D* in RA-differentiated cells from patient glioblastoma stem-like cells were analyzed by quantitative RT-PCR and shown as fold change over mRNA levels in patient glioblastoma stem-like cells. Error bars represent the standard deviation (*n* = 3). **c** RA-differentiated cells from CD1d-positive patient glioblastoma stem-like cells were pulsed overnight with α-GalCer (200 ng/ml) and co-cultured with resting iNKT cells for 4 h at a 10:1 ratio, and the cytotoxicity was also assessed by measuring LDH release. Error bars represent the SEM (*n* = 3). *P < 0.05

Glioblastoma contains cancer stem-like cells, which undergo self-renewal and are resistant to therapeutic approaches. We sought to determine if stem-like cells derived from glioblastoma also express CD1d to induce NKT cell-mediated anti-tumor responses. To address this question, we generated glioblastoma stem-like cells from patient glioblastoma and assessed CD1d expression levels by flow cytometry. The expression level of CD1d on stem-like cells derived from patient glioblastoma was lower than that on the original patient glioblastoma cells (Fig. 4a). We then assessed if CD1d expression on glioblastoma stem-like cells can be upregulated to induce iNKT cell-mediated anti-tumor responses effectively.

RA is the most common differentiating agent in clinical practice and all-trans RA has been successfully used to treat acute promyelocytic leukemia, a stem cell malignancy [22]. Previous work reported the transcriptional regulation of *CD1D* expression in human B cells by all-trans RA [23]. This led us to the hypothesis that RA may induce CD1d expression in glioblastoma stem-like cells. To test the hypothesis, we established RA-differentiated cells from both CD1d-positive and -negative stem-like cells of glioblastoma. The expression level of CD1d on RA-differentiated cells was ascertained to be higher than that on the glioblastoma stem-like cells (Fig. 4a). All-trans RA induced CD1d expression on glioblastoma stem-like cells. Consistent with this notion, RT-PCR was used to examine the effects of all-trans RA on CD1d expression in glioblastoma stem-like cells. The mRNA levels of the *CD1D* gene in RA-differentiated cells from patient glioblastoma stem-like cells were higher than the levels in patient glioblastoma stem-like cells, suggesting that CD1d expression was elevated (Fig. 4b).

### iNKT cells recognize RA-differentiated cells from CD1d-positive patient glioblastoma stem-like cells and show cytotoxicity

Next, to assess whether RA-differentiated cells were also sensitive to α-GalCer-dependent iNKT cell-mediated cytotoxicity, we performed an in vitro cytotoxicity assay using RA-differentiated cells from CD1d-positive patient glioblastoma stem-like cells from patient No. 22 or CD1d-negative patient glioblastoma stem-like cells from patient No. 29. iNKT cells exhibited higher cytotoxicity against α-GalCer-pulsed RA-differentiated cells from CD1d-positive patient glioblastoma stem-like cells than those without α-GalCer (Fig. 4c). In contrast, RA-differentiated cells from CD1d-negative patient glioblastoma stem-like cells had slightly higher sensitivity to α-GalCer-dependent iNKT cell-mediated cytotoxicity, though the difference was not statistically significant (Supplementary Fig. 1).

### iNKT cells recognize CD1d-transfected U87 cells and show cytotoxicity

**Fig. 5:**
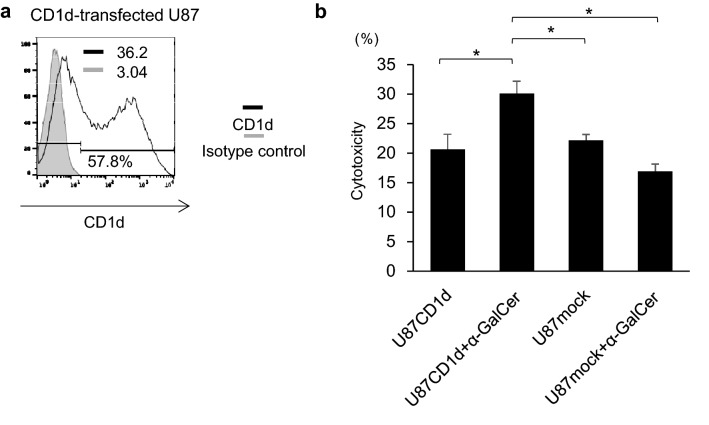
iNKT cells recognize CD1d-transfected U87 cells and show cytotoxicity. **a** The expression levels of CD1d in CD1d-transfected U87 cells were assessed by flow cytometry. Solid lines: staining profiles of CD1d. Shaded areas: background staining with an isotype control. **b** CD1d-transfected U87 cells were pulsed overnight with α-GalCer (200 ng/ml) and co-cultured with resting iNKT cells for 4 h at a 5:1 ratio. Cell apoptosis was evaluated by staining with annexin V and 7-AAD. Error bars represent the SEM (*n* = 3). *P < 0.05.

To assess if CD1d expression influences α-GalCer-dependent iNKT cell-mediated cytotoxicity, we used Lipofectamine 3000 to transiently transfect the CD1d-negative U87 cells with CD1d plasmid (Fig. 5a). We performed cytotoxicity assay with CD1d-transfected U87 cells. CD1d-transfected U87 cells were sensitive to α-GalCer-dependent iNKT cell-mediated cytotoxicity (Fig. 5b).

### Intracranial injection of human iNKT cells in an orthotopic xenogenic model of glioblastoma

iNKT cell-targeted therapies, such as autologous α-GalCer-pulsed antigen-presenting cells, are promising options for cancer treatment. However, because iNKT cells were not found in TILs from glioblastoma patients (Fig. 1e), iNKT cells needed to be introduced directly into patients’ brains. Therefore, we chose to undertake intracranial injections of purified iNKT cells in NOG mice. In cell cultures from human blood samples, it is feasible to expand functional iNKT cells with α-GalCer. We tested the therapeutic potential of iNKT cells against human glioblastoma cells in vivo using an orthotopic glioblastoma model in NOG mice.

**Fig. 6:**
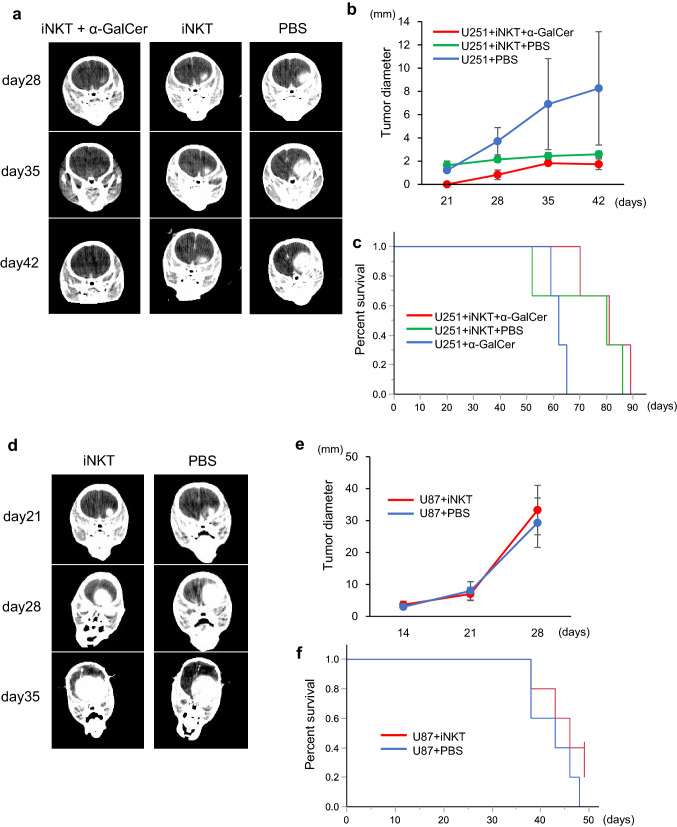
Anti-tumor activity of intracranial transfer of iNKT cells in an orthotopic glioblastoma model. **a** U251 cells were injected into the mouse brain as described in the “Materials and methods”. iNKT cells with α-GalCer (200 ng/mouse), iNKT cells alone, or PBS were injected intracranially on the same day. Representative CT images at the indicated time intervals are shown. **b** The graph represents tumor progression in the indicated groups as measured in **a**. Data are from three mice per group and shown as mean ± SEM. **c** U251 cells were intracranially injected with iNKT cells and α-GalCer (200 ng/mouse), iNKT cells alone, or α-GalCer (200 ng/mouse) alone on the same day. Survival rates in the indicated groups of mice were evaluated. *P* < 0.05: α-GalCer alone vs. iNKT cells with α-GalCer. **d** U87 cells were injected into mouse brain as described in the “Materials and methods”. iNKT cells or PBS were injected intracranially on the same day. Representative CT images at the indicated time intervals are shown. **e** The graph represents tumor progression in the indicated groups as measured in **d**. Data are from three mice per group and shown as mean ± SEM. **f** Survival rates in the indicated groups of mice were evaluated

CD1d-positive U251 cells were intracranially injected with iNKT cells and α-GalCer into mice, and tumor growth was monitored by micro-CT (Fig. 6a). iNKT cell injection with or without α-GalCer tended to delay tumor growth compared with the control injection, though the difference was not statistically significant (Fig. 6b). Furthermore, the co-injection of iNKT cells with α-GalCer significantly extended the survival of tumor-bearing mice compared with α-GalCer alone (*P* < 0.05) (Fig. 6c). No significant difference was observed in survival between intracranial injection of iNKT cells alone and α-GalCer alone (Fig. 6c).

In contrast, iNKT cells failed to repress tumor growth of CD1d-negative U87 cells in the intracranial injection model (Fig. 6d–f).

**Fig. 7:**
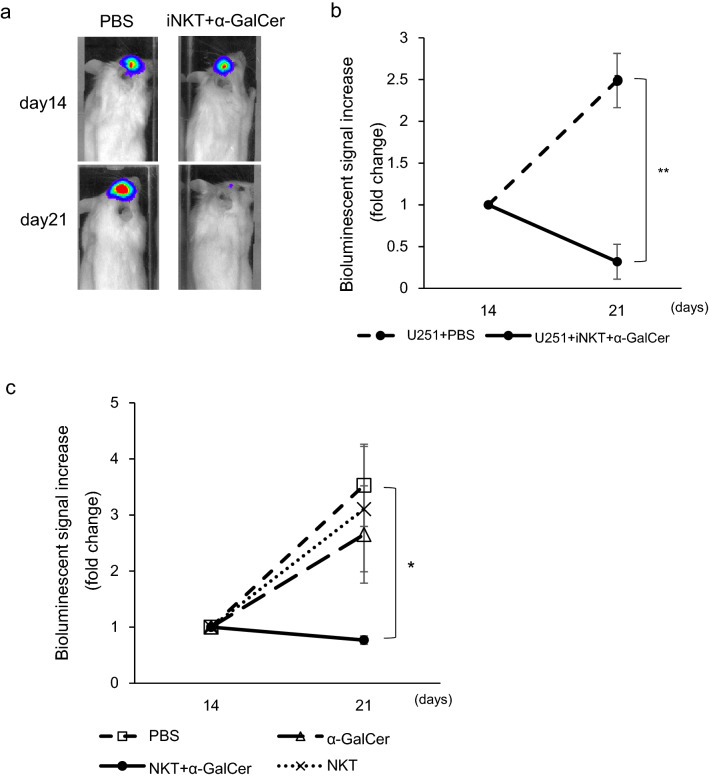
Intracranial administration of iNKT cells has therapeutic effects on established tumors in an orthotopic glioblastoma model. **a** Luciferase-transduced U251 cells were injected into the mouse brain as described in the “Materials and methods”. iNKT cells with α-GalCer (200 ng/mouse) or PBS were injected intracranially on day 7. Representative images at indicated time intervals are shown. **b** Tumor progression in the indicated groups was measured by bioluminescence (BL) signal intensity. Data are from 4–5 mice per group and shown as mean ± SEM. ^**^P < 0.005. **c** NKT cells with α-GalCer (200 ng/mouse), iNKT cells alone, α-GalCer alone, or PBS were injected intracranially on day 7. Tumor progression in the indicated groups was measured by BL signal intensity, and was analyzed as described in **b**. Data are from five mice per group and shown as mean ± SEM. ^*^P < 0.05

Finally, we tested the impact of iNKT cell treatment on established glioblastoma. Luciferase-transduced U251 cells were intracranially injected. After confirmation of tumor engraftment on day 6, mice were treated with intracranial injections of iNKT cells with α-GalCer or an equal volume of vehicle control on day 7 and were monitored for tumor burden by weekly bioluminescent imaging. A significantly faster decline in tumor burden was observed within 2 weeks after the injection of iNKT cells with α-GalCer (Fig. 7a, b). We also treated the xenograft mice with intracranial injections of iNKT cells with α-GalCer, iNKT cells alone, α-GalCer alone, or vehicle control on day 7 and monitored them for tumor burden by weekly bioluminescent imaging. A significantly faster decline in tumor burden was observed within 2 weeks after the injection of iNKT cells with α-GalCer (Fig. 7c). In contrast, iNKT cells alone and α-GalCer alone failed to repress tumor growth.

Therefore, CD1d expression in glioblastoma is a promising target for NKT cell-based cancer immunotherapy.

## Discussion

CD1d is a glycolipid antigen-presenting molecule recognized by iNKT cells. Although the majority of solid tumors are CD1d-negative, several studies have reported the CD1d expression in malignant tumors including glioblastoma [17–20]. Moreover, Dhodapkar et al. demonstrated that both the frequency and function of iNKT cells were well preserved in adult patients with glioma [20]. However, the functional role of tumor-infiltrating iNKT cells in glioblastoma has not yet been determined.

Dhodapkar et al. also demonstrated that the expression of CD1d is detected both on primary glioma cells and endothelial cells in infiltrating new blood vessels through immunohistochemistry of glioma tissue sections [20]. On the other hand, our data demonstrated patient glioblastoma cells express CD1d on the cell surface by flow cytometry. Since we excluded hematopoietic cells, which should contain CD1d-positive cell populations, by density gradient centrifugation in Lymphocyte Separation Media and CD45 staining, the CD1d-expressing cells were glioblastoma cells. Furthermore, the CD1d molecule in glioblastoma was functional, as CD1d-positive glioblastoma cells became highly sensitive to direct iNKT cell-mediated cytotoxicity after treatment with α-GalCer in vitro.

TILs are found in a variety of cancers and are considered as prognostic factors and predictors of therapeutic efficacy [24–26]. iNKT cells in TILs have also been reported to be a positive prognostic factor for colorectal carcinoma [27]. In glioblastoma lesions, several reports have shown the presence of iNKT cells [28, 29], but the extent of iNKT cell infiltration into the glioblastoma microenvironment was undetectable in our report. This observation suggested that specific treatment methods of intracranial introduction of iNKT cells are required for glioblastoma patients. These adoptive immunotherapies remove the need for antigen presentation and stimulation of a primary immune response and are potentially more effective and could have more favorable responses compared with other immunotherapies. Especially, because iNKT cells exhibit direct cytotoxicity against CD1d-expressing glioblastoma cells, we propose that the adoptive immunotherapy of iNKT cells should be an ideal therapeutic strategy for glioblastoma.

Several clinical trials of NKT cell-targeted immunotherapy have shown promising results for non-small cell lung cancer and head and neck squamous cell carcinoma [15]. However, intracranial injection of ex vivo-expanded human iNKT cells showed little cytotoxicity for CD1d-negative glioblastoma cells in vivo. In contrast, we demonstrated that iNKT cells showed therapeutic activity against CD1d-positive glioblastoma xenografts. Thus, CD1d expression may represent a novel target for NKT cell-based cancer immunotherapy for glioblastoma patients.

Although iNKT cells had little in vitro cytotoxicity against glioblastoma cells in the absence of α-GalCer (Fig. 3a), in vivo treatment with iNKT cells alone tended to suppress tumor growth and extend survival (Fig. 6a–c). These results suggested that glioblastoma cells cross-present endogenous glycolipids in the brain. Liu et al. also indicated the presence of the endogenous glycolipids in the brain and reported that brain tissues are rich in glycosphingolipids including β-GlcCer, the main endogenous ligand for NKT cells [35]. Therefore, we suggest iNKT cell monotherapy would have a potential to exert anti-tumor function. The co-administration of iNKT cells and α-GalCer would be needed to enhance the therapeutic effects of iNKT cells (Fig. 7c).

Conventional radiotherapy and chemotherapy for glioblastoma appear to mainly target the most proliferative cancer cells and spare the low-proliferative neoplastic glioblastoma stem-like cells that are relatively resistant to current cytotoxic therapeutics [30–32]. It is postulated that targeting glioblastoma stem-like cells will be more effective [33]. Interestingly, in glioblastoma stem-like cells, the expression levels of CD1d were lower than the expression levels of the CD1d-positive patient glioblastoma cells. However, CD1d expression was ascertained in FBS-differentiated glioblastoma cells from CD1d-positive patient glioblastoma cells (Fig. 2c). These findings raise the possibility that CD1d expression was induced on the surface of stem-like cells by differentiation.

RA is the most common differentiating agent in clinical practice and all-trans RA has become the first-choice drug for the treatment of acute promyelocytic leukemia [34]. RA also potently inhibits glioblastoma stem-like cell proliferation and induces stem-like cell differentiation [23]. In the current study, we found that CD1d expression on glioblastoma stem-like cells from patient tissue was increased after stimulation with all-trans RA. Moreover, RA-differentiated cells from CD1d-positive patient glioblastoma were also sensitive to α-GalCer-dependent iNKT cell-mediated cytotoxicity. Thus, the combination of RA treatment and iNKT cell-based therapies may benefit patients with CD1d-positive glioblastoma.

In conclusion, these findings suggest that CD1d expression in glioblastoma should be a critical determinant for NKT cell therapy. It is reasonable to speculate that precision medicine determined by CD1d expression in glioblastoma is required for NKT cell-based cancer immunotherapy. Further studies in vitro and in vivo are warranted to clarify the underlying mechanisms and test the feasibility of this new therapy.

## Electronic supplementary material

Below is the link to the electronic supplementary material.

Supplementary file1 (PPTX 43 kb)

## Data Availability

Derived data supporting the findings of this study are available from the corresponding author on request.

## Abbreviations

α-GalCer: α-Galactosylceramide
E:T: Effector-to-target
IFNγ: Interferon-γ
iNKT: Invariant natural killer T
LDH: Lactate dehydrogenase
MFI: Median fluorescence intensity
NOG: NOD/Shi-scid IL-2RγKO
PBMCs: Peripheral blood mononuclear cells
RA: Retinoic acid
RANO: Response assessment in neuro-oncology
SEM: Standard error of the mean
TCR: T cell receptor
TILs: Tumor-infiltrating lymphocytes
TNFα: Tumor necrosis factor-α
