# Supplementary material for: CD1d expression in glioblastoma is a promising target for NKT cell-based cancer immunotherapy
Source: Cancer Immunol Immunother. 2020 Oct 31;70(5):1239–54. doi: 10.1007/s00262-020-02742-1 (PMC8053161; doi:10.1007/s00262-020-02742-1)
Supplement: Supplementary file 1 — Supplementary file1 (PPTX 43 kb) [file 262_2020_2742_MOESM1_ESM.pptx]

## Slide 1
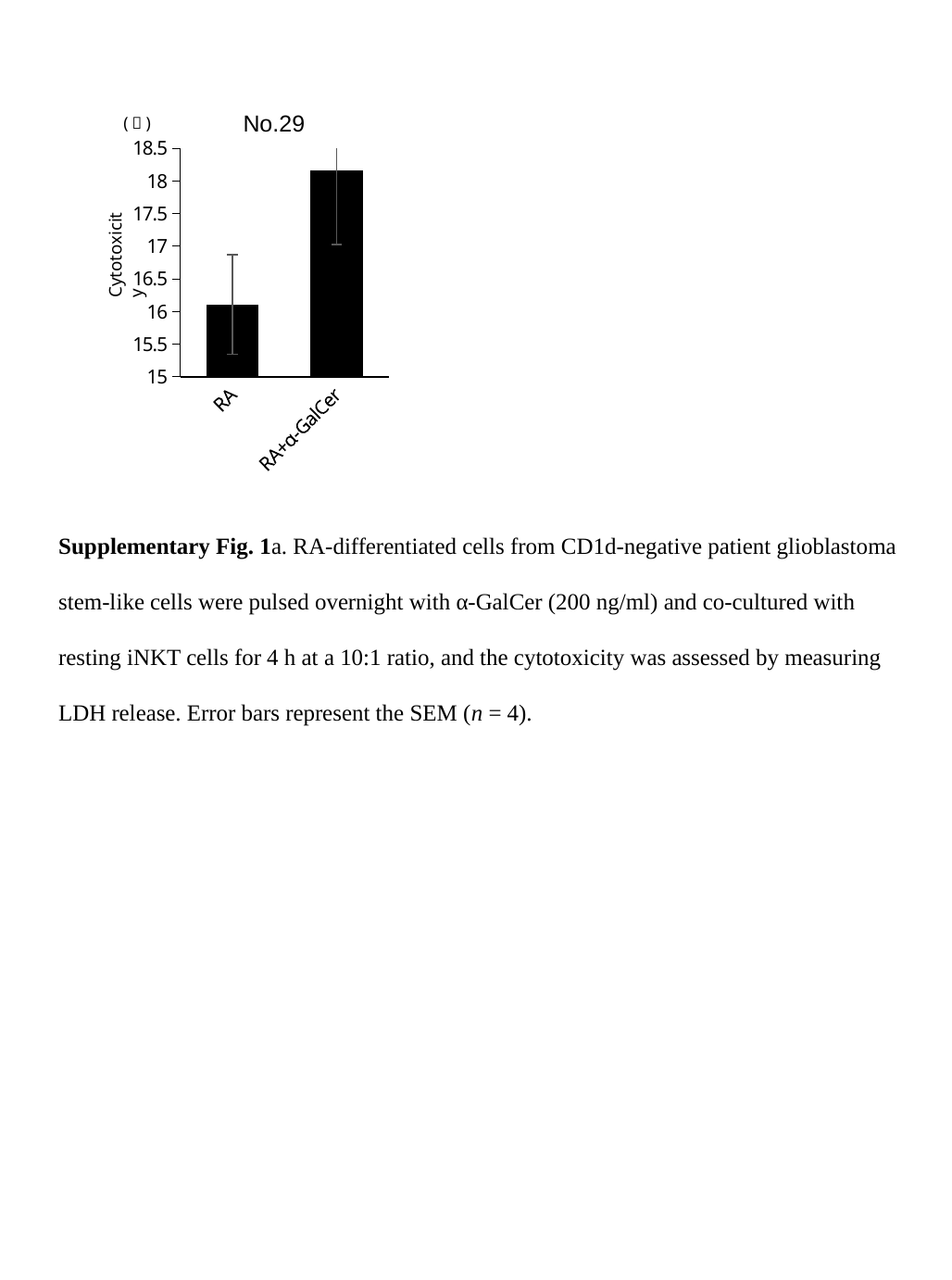

(％)
No.29
### Chart
| Category | |
|---|---|
| RA | 16.107394123327545 |
| RA+α-GalCer | 18.16327719829844 |Cytotoxicity
Supplementary Fig. 1a. RA-differentiated cells from CD1d-negative patient glioblastoma stem-like cells were pulsed overnight with α-GalCer (200 ng/ml) and co-cultured with resting iNKT cells for 4 h at a 10:1 ratio, and the cytotoxicity was assessed by measuring LDH release. Error bars represent the SEM (n = 4).
